# Training Medical Students/Medical Practitioners to Diagnose Dermatologic Conditions in Skin of Color: A Systematic Review of Educational Interventions

**DOI:** 10.1177/12034754251408403

**Published:** 2026-01-22

**Authors:** Andy D. Lee, Amrit Thandi, Jinny Choi, Grace Xiong, Sara Pollanen, Kristen Amanda Rachel Yee, Fatemeh Jafarian, Cathryn Sibbald

**Affiliations:** 1 Temerty Faculty of Medicine, University of Toronto, Toronto, ON, Canada; 2 Michael G.DeGroote School of Medicine, McMaster University, Hamilton, ON, Canada; 3 Faculty of Medicine & Dentistry, University of Alberta, Edmonton, AB, Canada; 4 Division of Dermatology, Department of Medicine, University of Calgary, Calgary, Canada; 5 Division of Pediatric Dermatology, The Hospital for Sick Children, University of Toronto, Toronto, ON, Canada

**Keywords:** skin of color, dermatology education, medical curriculum, diagnostic disparities, cultural competence

## Abstract

Despite increasing recognition of the importance of skin-of-color (SOC) representation in dermatology education, significant gaps persist in training medical students to diagnose dermatologic conditions across diverse skin tones. This systematic review analyzed educational interventions aimed at improving SOC diagnostic competency among medical trainees. A comprehensive search of MEDLINE, Embase, APA PsycInfo, and Scopus identified 8 studies comprising 667 participants, revealing categories: didactic teaching (33.1%), seminar-style expert-led teaching (32.4%), workshop-based learning (25.8%), and simulation-based learning (8.7%). Preintervention assessments consistently showed significantly lower confidence in diagnosing conditions in darker skin tones, with 64% to 69% of students reporting no SOC training at undergraduate level. All interventions produced statistically significant improvements in self-assessed knowledge and confidence, though 87.2% of students still lacked confidence postintervention. The most promising approach was Hardin’s two-week combined training protocol, which mitigated initial diagnostic bias between light and dark skin diagnoses (*P* = .007). Critical limitations include predominantly single-session interventions, reliance on self-reported confidence measures, absence of long-term retention data, and persistent condition-specific diagnostic challenges, particularly for melanoma in darker skin. Addressing these educational gaps represents both an imperative for medical education reform and a crucial step toward achieving health equity in dermatologic care.

## Introduction

The underrepresentation of skin-of-color (SOC) in dermatology education remains a critical barrier to equitable healthcare delivery, contributing to delayed diagnoses, mismanagement, and poorer outcomes for patients with darker skin tones.^1^ While awareness of this disparity has grown substantially over the past decade, systematic approaches to address these educational gaps remain fragmented and understudied. This systematic review synthesizes current evidence on educational interventions designed to improve medical students’ and practitioners’ ability to diagnose dermatologic conditions across diverse skin tones.

## Methods

A systematic search of MEDLINE, Embase, APA PsycInfo, and Scopus was conducted on 26 December 2024 following PRISMA guidelines (PROSPERO: CRD42025636964). Search terms combined “medical students,” “dermatology education,” and “skin of color” with relevant synonyms (Supplemental Figure S1; Supplemental Table S1). Inclusion criteria encompassed studies evaluating educational interventions targeting SOC dermatology competency among medical students, residents, or early-career physicians. Data extracted included intervention types, assessment methods, participant demographics, and outcomes (Supplemental Table S2). Study quality was assessed using the Oxford Centre for Evidence-Based Medicine 2011 Levels of Evidence.

## Results

### Study Characteristics and Participant Demographics

Our search identified 8 studies meeting inclusion criteria, collectively evaluating 667 participants across diverse training levels. The cohort included first-year medical students (n = 199), second-year students (n = 58), fourth-year students (n = 158), mixed-year medical students (n = 177), and junior doctors/residents (n = 75). Studies were conducted primarily in the United States (n = 5), with additional contributions from Germany (n = 1), the United Kingdom (n = 1), and Canada (n = 1).

### Intervention Categories and Delivery Methods

Analysis revealed 4 distinct pedagogical approaches across the included studies (Supplemental Table S3):

Didactic Teaching (33.1%, 221/667): Traditional lecture-based curricula represented the most common intervention type. Hijab et al.’s single-week curriculum revision reached 107 first-year medical students, producing statistically significant improvements in viewing dermatology as a culturally competent specialty (*P* < .001) and increased specialty interest (*P* = .048).^1^ Peterknecht’s 60-minute interactive lecture combined traditional didactic elements with game-based activities, yielding confidence improvements of 2.5 and 1.82 points for medical students and junior doctors, respectively (*P* < .001).^2^
Seminar-Style Expert-Led Teaching (32.4%, 216/667): Expert-facilitated seminars emphasized interactive discussion and case-based learning. Arza et al.’s virtual dermatologist-led seminar highlighted persistent gaps, with 87.2% of participants lacking confidence in SOC diagnosis even postintervention.^5^ Conversely, Abeck’s mandatory 90-minute seminar incorporating psychosocial burden discussions demonstrated robust improvements in self-assessed knowledge (2.9 ± 1.3 to 4.5 ± 0.9, *P* < .001) and diagnostic confidence (2.3 ± 1.2 to 3.9 ± 1.1, *P* < .001).^3^
Interactive/Workshop-Based Learning (25.8%, 172/667): Thompson’s 75-minute multimodal workshop achieved significant confidence improvements across all learning objectives, with melanoma recognition confidence increasing from 1.0 to 4.0 on a 5-point scale.^7^ Hardin’s innovative 2-week protocol combining nonanalytic training with skin tone-specific patient cards and targeted workshops demonstrated the most robust objective outcomes, eliminating diagnostic performance bias between light and dark skin cases (*P* = .007).^6^
Simulation-Based Learning (8.7%, 58/667): Robinson’s opportunistic screening exercise using melanoma moulages revealed concerning baseline performance, with only 16% of students detecting the lesion. While detection rates were equal between Black and white patients, students were significantly more likely to suggest melanoma as a diagnosis in white patients (*P* = .03).^4^

### Preintervention Baseline Assessment

Baseline assessments revealed alarming educational deficits across all studies. Peterknecht reported that 64% of medical students and 69% of junior doctors had received no SOC dermatology training at the undergraduate level, with 84% of junior doctors reporting no postgraduate training.^2^ Students consistently reported significantly lower confidence diagnosing conditions in darker skin tones compared to lighter skin, with mean confidence differentials ranging from 0.8 to 2.5 points on 5-point scales. Qualitative assessments universally identified Eurocentric bias in educational materials, with textbook representations featuring light skin images at approximately 5:1 ratios compared to darker skin tones.^6^

### Intervention Outcomes and Efficacy

All studies demonstrated statistically significant improvements in at least one measured outcome domain:

Knowledge Gains: Objective knowledge assessments, utilized in only three studies, showed variable improvements. Peterknecht’s eight-question assessment scores increased by 3.68 and 3.87 points for medical students and junior doctors, respectively (*P* < .001).^2^ Hardin’s diagnostic accuracy measurements revealed initial performance deterioration on SOC cases following nonanalytic training alone (*P* = .0002), but subsequent combined training eliminated this bias (*P* = .007).^6^
Confidence and Self-Efficacy: Self-reported confidence improvements were universal but varied in magnitude. Abeck reported the largest absolute gains, with diagnostic confidence increasing by 1.6 points on a 6-point scale.^3^ However, Arza found that 87.2% of students still lacked confidence postintervention, with 78.7% attributing persistent deficits to inadequate curriculum coverage.^5^
Cultural Competence: Studies measuring cultural competence dimensions showed promising results. Students demonstrated increased awareness of psychosocial burdens specific to SOC populations, including postinflammatory hyperpigmentation visibility and vitiligo impact (pre: 3.2 ± 1.3, post: 4.7 ± 0.9, *P* < .001).^3^

### Condition-Specific Performance

Melanoma diagnosis in darker skin emerged as a particularly recalcitrant challenge. Despite targeted interventions, diagnostic accuracy for melanoma on SOC remained significantly lower than on light skin across multiple studies. Robinson’s moulage study revealed not only low overall detection rates (16%) but also diagnostic reasoning disparities.^4^ Hardin similarly found that while overall diagnostic bias was eliminated post-intervention, melanoma-specific performance gaps persisted.^6^

### Long-Term Retention and Sustainability

Critical gaps in long-term outcome assessment limit conclusions about intervention durability. Only Hardin’s study included any follow-up assessment, with a poststudy survey suggesting skill retention, though specific timeframes and objective measures were not reported.^6^ The predominance of single-session interventions (75% of studies) raises concerns about knowledge decay and sustainability of confidence improvements.

### Qualitative Findings and Student Perspectives

Thematic analysis of qualitative feedback revealed consistent patterns across studies. Students universally requested increased SOC representation in educational materials, with 82.9% in Abeck’s study desiring additional dedicated courses.^3^ Participants highlighted appreciation for interactive and multimodal learning approaches. Structural barriers identified included limited faculty diversity, inadequate clinical exposure to diverse patient populations, and the absence of SOC considerations in standardized assessments. Many students reported being unaware of diagnostic challenges in SOC before participating in interventions.^6^

## Discussion

This systematic review reveals both encouraging progress and persistent challenges in addressing SOC diagnostic competency gaps in medical education. While all evaluated interventions produced measurable improvements, the magnitude and durability of these gains remain insufficient to ensure diagnostic equity across skin tones.

The heterogeneity of intervention approaches reflects the absence of evidence-based best practices in SOC dermatology education. Single-session interventions, while logistically feasible, appear inadequate for developing sustained competency. Hardin’s multiweek combined training protocol, integrating both pattern recognition and rule-based approaches, offers a promising model for more comprehensive curriculum development.^6^ The initial performance deterioration observed with nonanalytic training alone underscores the complexity of recalibrating visual diagnostic skills.

The persistent challenge of melanoma diagnosis in darker skin warrants particular attention. Given melanoma’s potential lethality and documented survival disparities between racial groups, targeted interventions addressing this specific diagnostic challenge should be prioritized. Thompson’s melanoma-focused workshop provides a foundation, but integration with longitudinal reinforcement and objective performance assessment remains necessary.^7^

Our findings align with broader calls for structural reform in medical education.^8^ The revelation that 64-69% of trainees receive no formal SOC education at the undergraduate level represents a systemic failure requiring institutional-level intervention.^2^ Beyond individual teaching sessions, comprehensive reform must address textbook representation, clinical rotation diversity, assessment standards, and faculty development.

Several limitations constrain the generalizability of our findings. The predominance of single-institution studies with small sample sizes limits statistical power and external validity. Reliance on self-reported confidence measures may not correlate with actual diagnostic performance. The absence of patient outcome data prevents assessment of clinical impact.

### Recommendations and Future Directions

Based on our systematic analysis, we propose the following evidence-informed recommendations:

1. Longitudinal Integration: Replace single-session interventions with longitudinal curricula spanning preclinical and clinical years, with regular reinforcement and progressive complexity.
2. Objective Assessment: Develop validated tools for measuring diagnostic accuracy across skin tones, moving beyond self-reported confidence to performance-based metrics.
3. Condition-Specific Modules: Create targeted interventions for high-stakes diagnoses like melanoma, with emphasis on visual pattern recognition and clinical reasoning skills.
4. Structural Reform: Advocate for institutional changes including diverse image databases, inclusive textbook adoption criteria, and recruitment of faculty with SOC expertise.
5. Research Priorities: Conduct multicenter randomized trials comparing intervention modalities, assess long-term retention, and evaluate impact on patient outcomes.

## Conclusion

Addressing diagnostic disparities in dermatology education represents both a moral imperative and a practical necessity in increasingly diverse societies. While current interventions demonstrate the feasibility of improving SOC diagnostic competency, transformative change requires moving beyond isolated workshops to comprehensive curriculum reform. The persistent postintervention confidence gaps and condition-specific challenges identified in this review underscore that achieving diagnostic equity demands sustained institutional commitment, evidence-based pedagogical innovation, and ongoing assessment of both educational and clinical outcomes. As medical education evolves to meet 21st-century demographic realities, ensuring diagnostic competency across all skin tones must transition from optional enhancement to core curricular requirement.

## Supplemental Material

sj-docx-1-cms-10.1177_12034754251408403 – Supplemental material for Training Medical Students/Medical Practitioners to Diagnose Dermatologic Conditions in Skin of Color: A Systematic Review of Educational Interventions Supplemental material, sj-docx-1-cms-10.1177_12034754251408403 for Training Medical Students/Medical Practitioners to Diagnose Dermatologic Conditions in Skin of Color: A Systematic Review of Educational Interventions by Andy D. Lee, Amrit Thandi, Jinny Choi, Grace Xiong, Sara Pollenan, Kristen Amanda Rachel Yee, Fatemeh Jafarian and Cathryn Sibbald in Journal of Cutaneous Medicine and Surgery

sj-docx-2-cms-10.1177_12034754251408403 – Supplemental material for Training Medical Students/Medical Practitioners to Diagnose Dermatologic Conditions in Skin of Color: A Systematic Review of Educational Interventions Supplemental material, sj-docx-2-cms-10.1177_12034754251408403 for Training Medical Students/Medical Practitioners to Diagnose Dermatologic Conditions in Skin of Color: A Systematic Review of Educational Interventions by Andy D. Lee, Amrit Thandi, Jinny Choi, Grace Xiong, Sara Pollenan, Kristen Amanda Rachel Yee, Fatemeh Jafarian and Cathryn Sibbald in Journal of Cutaneous Medicine and Surgery

sj-docx-3-cms-10.1177_12034754251408403 – Supplemental material for Training Medical Students/Medical Practitioners to Diagnose Dermatologic Conditions in Skin of Color: A Systematic Review of Educational Interventions Supplemental material, sj-docx-3-cms-10.1177_12034754251408403 for Training Medical Students/Medical Practitioners to Diagnose Dermatologic Conditions in Skin of Color: A Systematic Review of Educational Interventions by Andy D. Lee, Amrit Thandi, Jinny Choi, Grace Xiong, Sara Pollenan, Kristen Amanda Rachel Yee, Fatemeh Jafarian and Cathryn Sibbald in Journal of Cutaneous Medicine and Surgery

sj-docx-4-cms-10.1177_12034754251408403 – Supplemental material for Training Medical Students/Medical Practitioners to Diagnose Dermatologic Conditions in Skin of Color: A Systematic Review of Educational Interventions Supplemental material, sj-docx-4-cms-10.1177_12034754251408403 for Training Medical Students/Medical Practitioners to Diagnose Dermatologic Conditions in Skin of Color: A Systematic Review of Educational Interventions by Andy D. Lee, Amrit Thandi, Jinny Choi, Grace Xiong, Sara Pollenan, Kristen Amanda Rachel Yee, Fatemeh Jafarian and Cathryn Sibbald in Journal of Cutaneous Medicine and Surgery
