# Supplementary material for: Training Medical Students/Medical Practitioners to Diagnose Dermatologic Conditions in Skin of Color: A Systematic Review of Educational Interventions
Source: J Cutan Med Surg. 2026 Jan 22;30(4):405–8. doi: 10.1177/12034754251408403 (PMC13478447; doi:10.1177/12034754251408403)
Supplement: Supplementary file 1 [file sj-docx-1-cms-10.1177_12034754251408403.docx]

**28** studies assessed for eligibility on basis of full-text

**20** studies excluded:

**15** No interventions

**2** Does not have any outcomes

**2** Not about medical/ healthcare professional students

**1** is conference paper

**25** studies included

**Screening**

**Included**

**133** studies screened on basis of title and abstract

**101** studies irrelevant

**168** studies identified from:

MEDLINE

Embase

APA PsycInfo

Cochrane Central

Scopus

Pubmed

Studies removed before screening:

**35** duplicates removed

**Identification of studies via databases and registers**

**Identification**

The criteria for study inclusion were: i) Studies involving medical students or healthcare students, ii) intervention/ modules addressing skin of color in dermatology, iii) at least one learning outcome or measure

**Supplementary Figure 1.** Flow diagram of literature screening using the Preferred Reporting Items for Systematic Reviews and Meta-Analyses guidelines. Figure adapted from http://prisma-statement.org.
