# Supplementary material for: Training Medical Students/Medical Practitioners to Diagnose Dermatologic Conditions in Skin of Color: A Systematic Review of Educational Interventions
Source: J Cutan Med Surg. 2026 Jan 22;30(4):405–8. doi: 10.1177/12034754251408403 (PMC13478447; doi:10.1177/12034754251408403)
Supplement: Supplementary file 2 [file sj-docx-2-cms-10.1177_12034754251408403.docx]

Supplementary Table 1. Ovid MEDLINE(R) and Embase Classic and PSYCInfo ALL <1946 to Dec 26, 2024>

| **#** | **Search line** | **Results** |
| --- | --- | --- |
| 1 | medical student/  OR ( (medical student* OR “medical trainee*” OR "undergraduate medical student*"  OR “undergraduate medical education” OR UGME OR MBBS OR “MD program” )  ADJ (education OR curriculum OR program*) ).mp.  OR (medical student* OR “undergraduate medical education” OR UGME OR MBBS  OR “MD program” OR BMBS OR “bachelor of medicine” OR “bachelor of surgery”).mp. | 224177 |
| 2 | exp undergraduate education/  OR ( (undergraduate OR predoctoral) ADJ (education OR curriculum OR training OR teaching) ).mp.  OR ("medical curriculum" OR "medical teaching" OR "undergraduate teaching"  OR "undergraduate curriculum").mp. | 34369 |
| 3 | 1 OR 2 | 247942 |
| 4 | dermatology/ed OR dermatology.fs. OR (dermatology adj3 (training OR education OR curriculum OR teaching OR intervention OR program OR module OR course OR workshop OR instruction)).mp. | 2880 |
| 5 | skin pigmentation/  OR ( "skin of color" OR "darker skin" OR "dark complexion"  OR "Fitzpatrick IV" OR "Fitzpatrick V" OR "Fitzpatrick VI"  OR "Fitzpatrick 4" OR "Fitzpatrick 5" OR "Fitzpatrick 6"  OR "dermatologic diversity" OR "skin tone diversity"  OR "skin color" OR "cutaneous diversity"  OR "pigmented skin" ).mp. | 49352 |
| 6 | cultural diversity/ OR minority group/ OR racial disparit* OR ethnic disparit* OR rac* adj disparit* OR minorit* OR "marginalized communit*" OR "BIPOC" OR "Black Indigenous People of Color" OR "African American*" OR "Hispanic*" OR "Latinx" OR "Southeast Asian*" OR "First Nations" OR "Aboriginal" OR "Indigenous population*" OR "Native American*".mp. | 927778 |
| 7 | 5 OR 6 | 973850 |
| 8 | empathy/  OR cultural competence/  OR clinical competence/  OR empath* OR compassion* OR “cultural competenc*” OR “cultural humility”  OR “cultural safety” OR “patient-centered” OR “patient-centred”  OR humanism OR “humanistic skill*”  OR OSCE OR “objective structured clinical exam*”  OR training adj outcome* OR learning adj outcome*  OR “educational outcome*” OR “clinical skill assessment”  OR “clinical competence” OR "bedside manner"  OR “rapport”.mp. | 264805 |
| 9 | 3 AND 4 AND 7 AND 8 | 8 |

Scopus

Inception to Dec 26th, 2024

| 1 | ( ( "medical student*" OR "medical trainee*" OR "undergraduate medical student*" OR "undergraduate medical education" OR ugme OR mbbs OR "MD program" OR bmbs OR "bachelor of medicine" OR "bachelor of surgery" ) OR ( ( undergraduate OR predoctoral ) W/3 ( education OR curriculum OR training OR teaching ) OR "medical curriculum" OR "medical teaching" OR "undergraduate teaching" OR "undergraduate curriculum" ) ) AND ( dermatolog* W/3 ( training OR education OR curriculum OR teaching OR intervention OR program OR module OR course OR workshop OR instruction ) ) AND ( ( "skin of color" OR "darker skin" OR "dark complexion" OR "Fitzpatrick IV" OR "Fitzpatrick V" OR "Fitzpatrick VI" OR "Fitzpatrick 4" OR "Fitzpatrick 5" OR "Fitzpatrick 6" OR "dermatologic diversity" OR "skin tone diversity" OR "skin color" OR "cutaneous diversity" OR "pigmented skin" ) OR ( "cultural diversity" OR "minority group" OR "racial disparit*" OR "ethnic disparit*" OR ( rac* W/3 disparit* ) OR minorit* OR "marginalized communit*" OR bipoc OR "Black Indigenous People of Color" OR "African American*" OR "Hispanic*" OR "Latinx" OR "Southeast Asian*" OR "First Nations" OR aboriginal OR "Indigenous population*" OR "Native American*" ) ) AND ( empath* OR compassion* OR "cultural competenc*" OR "cultural humility" OR "cultural safety" OR "patient-centered" OR "patient-centred" OR humanism OR "humanistic skill*" OR osce OR "objective structured clinical exam*" OR ( training W/3 outcome* ) OR ( learning W/3 outcome* ) OR "educational outcome*" OR "clinical skill assessment" OR "clinical competence" OR "bedside manner" OR rapport ) | 116 |
| --- | --- | --- |

Pubmed - 24 searches

(

("medical student*" OR "medical trainee*" OR "undergraduate medical student*"

OR "undergraduate medical education" OR UGME OR MBBS OR "MD program"

OR BMBS OR "bachelor of medicine" OR "bachelor of surgery")

OR

(

(undergraduate OR predoctoral) AND (education OR curriculum OR training OR teaching)

)

OR

("medical curriculum" OR "medical teaching" OR "undergraduate teaching" OR "undergraduate curriculum")

)

AND

(

dermatolog* AND

(training OR education OR curriculum OR teaching OR intervention

OR program OR module OR course OR workshop OR instruction)

)

AND

(

("skin of color" OR "darker skin" OR "dark complexion"

OR "Fitzpatrick IV" OR "Fitzpatrick V" OR "Fitzpatrick VI"

OR "Fitzpatrick 4" OR "Fitzpatrick 5" OR "Fitzpatrick 6"

OR "dermatologic diversity" OR "skin tone diversity"

OR "skin color" OR "cutaneous diversity"

OR "pigmented skin")

OR

("cultural diversity" OR "minority group" OR "racial disparit*"

OR "ethnic disparit*" OR (rac* AND disparit*)

OR minorit* OR "marginalized communit*" OR BIPOC

OR "Black Indigenous People of Color"

OR "African American*" OR "Hispanic*"

OR "Latinx" OR "Southeast Asian*"

OR "First Nations" OR aboriginal

OR "Indigenous population*"

OR "Native American*")

)

AND

(

empath* OR compassion*

OR "cultural competenc*"

OR "cultural humility"

OR "cultural safety"

OR "patient-centered"

OR "patient-centred"

OR humanism

OR "humanistic skill*"

OR OSCE

OR "objective structured clinical exam*"

OR ("training" AND outcome*)

OR ("learning" AND outcome*)

OR "educational outcome*"

OR "clinical skill assessment"

OR "clinical competence"

OR "bedside manner"

OR rapport

)
