# Supplementary material for: Training Medical Students/Medical Practitioners to Diagnose Dermatologic Conditions in Skin of Color: A Systematic Review of Educational Interventions
Source: J Cutan Med Surg. 2026 Jan 22;30(4):405–8. doi: 10.1177/12034754251408403 (PMC13478447; doi:10.1177/12034754251408403)
Supplement: Supplementary file 3 [file sj-docx-3-cms-10.1177_12034754251408403.docx]

**Supplementary Table 2.** Outcomes of educational intervention in training medical students and allied health care professionals. Abbreviations: AEs, adverse events; BID, twice-daily; BIW, twice weekly; CAR, case report; CAS, case series; CR, complete resolution; F, female; GI, gastrointestinal; IV, intravenous; M, male; m, months; NOR, no response; NR, none reported; PO, oral; PR, partial resolution; PRN, as needed; QD, daily; QW, weekly; Q2W, every 2 weeks; TIW, thrice weekly; TOP, topical; w, weeks

| **Article** | **Study type (evidence level)** | **Sample size** | **Years in medical school (allied health workers)** | **Mean age, years** | **Sex (n)** | **Type of intervention (lecture-based, online module, simulation/OSCE, high-fidelity images, case-based discussion, etc.)** | **Duration/ Format (single workshop, multi-week module, repeated sessions)** | **Pre-existing Curriculum (standard curriculum, no intervention, pre-test baseline, etc.)** | **Knowledge Gains / Diagnostic Accuracy (pre-post test scores, OSCE pass rates, interpretation of images)** | **Cultural Competence / Empathy Measures (Jefferson Scale of Empathy, cultural competence inventories, Likert-scale surveys)** | **Confidence or Self-Efficacy (subjective assessments, surveys)** | **Key Results (Quantitative)** | **Long-term retention** | **Attrition or dropouts** | **Qualitative Finding (Any comments/ feedback of students)** | **Conclusions** |
| --- | --- | --- | --- | --- | --- | --- | --- | --- | --- | --- | --- | --- | --- | --- | --- | --- |
| Hijab, 2022 | Descriptive | 107 (pre-test: 88, post-test: 49) | First-year medical students | NR | NR | Lecture-based curriculum revision | Single lecture, part of a one-week course | Pre-test baseline assessment | Pre- and post-test surveys on cultural competence and dermatology knowledge | Likert-scale surveys measuring diversity and inclusion awareness | Self-reported increase in awareness and interest in dermatology | ; Statistically significant increase in recognizing dermatology's role in cultural competence (P<0.001), increase in interest in dermatology (P=0.048) | NR | NR | Students preferred learning about disease variations across populations | Emphasizing cultural humility and diversity in curriculum improved student perspectives on dermatology |
| Arza, 2024 | Quasi-experimental (survey-based, pre-post design) | 58 completed pre-seminar surveys, 64 completed post-seminar surveys | First- to fourth-year medical students | NR | NR | Virtual, dermatologist-led seminar on skin health equity | Single seminar session with pre- and post-surveys (1st years received multidisciplinary lectures, 2nd/3rd years received dermatology and multidisciplinary lectures) | Limited SOC representation in dermatology curriculum | Significant mean differences in confidence diagnosing conditions in lighter (MST 1–5) vs. darker skin (MST 6–10) (p < 0.009) from pre-post test scores. | Likert-scale survey assessing SOC educational adequacy | Post-seminar, 87.2% lacked confidence diagnosing SOC conditions; 78.7% attributed it to inadequate curriculum coverage | 90% reported lower confidence in diagnosing dermatologic conditions on darker skin compared to lighter skin | NR | 0 | Students criticized the Eurocentric bias in textbooks and lack of diverse faculty, calling for improved SOC representation in medical education | Study underscores the need for better SOC representation in curricula. Single institution + voluntary participation may introduce bias via limited statistical power + a formatting error in the survey precluded the post-survey data from being included in the analysis. |
| Thompson, 2023 | Quasi-experimental study (pre-post design, Kirkpatrick levels 1 and 2 evaluation) | 80 participants (63 nursing students, 11 medical students/residents, 6 medical faculty) | Medical students, residents, and nursing students | NR | 56 F  24 M | Interactive workshop (PowerPoint lecture, video-based reflection, case discussions) | Single 75-minute session | No prior curriculum, needs assessment revealed a gap in dermatology education | Pre-post test. Statistically significant increase in learners’ confidence in recognizing melanoma in darker skin tones (Wilcoxon matched-pair signed-rank test, P<0.05) | Likert-scale survey: 5-point scale (1 = no confidence, 5 = complete confidence). | Significant self-reported increase in confidence across all learning objectives, below: 1. Describe the structure and components of  the skin.  2. Describe the etiology and clinical  manifestation of melanoma and its  subtypes.  3. Recognize various melanoma  presentations, especially in darker skin  tones.  4. List melanoma prevention and treatment  options | For the objective “Recognize various melanoma presentations, especially in darker skin tones”, confidence rating went from 1.0 pre-workshop to 4.0 post-workshop. | NR | 9 did not complete both pre- and post- session questionnaires, making total of 71 participants that completed both | Students appreciated the interactive format; requested more diverse images and larger slide fonts; Learners commended the use of videos, reflection exercises,  and active interaction through the case discussion | Workshop effectively increased awareness, but long-term retention and behavioral changes were not assessed. sample was relatively small, with a large representation of  nursing students. The workshop is a brief intervention and may  not yield sustained confidence in meeting the learning objectives.  The workshop has not been designed to assess for changes  in knowledge or behavior, components critical to reducing  clinical outcomes. Nor has the workshop been trialed using a  variety of facilitator knowledge levels; In terms of our evaluation tool, did not  label each response on the 5-point scale, only the anchors |
| Robinson 2010 | Descriptive study (clinical observation, survey-based assessment) | 58 second-year medical students (out of 140 invited) | Second-year medical students | NR | NR | Opportunistic screening exercise during dermatology patient exams using moulages on patients with dermatologic conditions | Single clinical session following a 1-hour lecture on melanoma detection | Minimal dermatology exposure in standard curricula; a single melanoma detection lecture prior to study | Observational detection exercise. Nine students noticed the “melanoma” lesion (16%).  There was no significant difference due to patient race  in noticing the lesion: 4 students noticed it on the black  patient and 5 students on the non-Hispanic white patient. Students interested in dermatology were more likely  to notice the melanoma (P=.04). Students identifying the  lesion were more likely to offer melanoma as a possible  diagnosis in the non-Hispanic white patient than they were  in the black patient (P=.03) | NR | NR | Only 16% of students detected the melanoma moulage. Equal detection on Black vs. white patients, but students were more likely to suggest melanoma in white patients (p = 0.03). Students interested in dermatology were more likely to notice lesions (p = 0.04). | NR | 0 | Participants valued interactive and multi-modal learning. Requested more SOC images and better slide readability. | Study highlights disparities in melanoma recognition across skin tones; further dermatology training is needed for all medical students; The homogeneous study sample  of the study with overrepresentation of those interested  in dermatology limits the generalizability of conclusions drawn from this study |
| Hardin 2023 | Prospective randomized intervention study | 92 (out of 167 enrolled) | First-year medical students | NR | NR | Online non-analytic training (NAT) using skin-tone-specific patient cards followed by combined training (CT) with workshops on diagnostic rules for all skin tones | 2 weeks, non-analytic training in week 1, “several” workshops held in week 2. | Limited exposure to skin of color (SoC) in dermatology training; standard curricula overrepresent light skin (LS) images (~5:1 ratio) | After NAT, students performed worse on SoC cases (p = 0.0002 for LS-trained group, p = 0.002 for SoC-trained group); after CT, diagnostic performance bias disappeared (p = 0.0001 for LS-trained group, p = 0.007 for SoC-trained group) | Survey showed increased awareness of bias in SoC diagnosis and recognition of gaps in dermatology education | Students initially lacked confidence in diagnosing SoC conditions but reported improvement post-training | After CT, students' diagnostic performance on SoC cases became equal to LS cases; initial diagnostic bias was significantly reduced. Although the overall mean diagnostic performance on LS and SoC was similar after CT, there was still heterogeneity  between the individual skin conditions. For example, the success of students diagnosing melanoma on SoC increased, but was still noticeably  lower than diagnostic performance of melanoma on LS. | Post-study survey suggested retention of skills, but long-term data is limited. 5/5 found the curriculum enhanced their diagnostic skills  in SoC | 0 | Before starting the dermatology course, the majority of students did not realise that there were challenges related to diagnosing some skin conditions in SoC. | Study confirms that a skin-tone balanced dermatology curriculum mitigates performance bias, but long-term retention and broader curricular integration need further study; this was a single-centre  study assessing the performance of a single group of learners on a limited number of skin conditions, which reduces the generalisability of  findings. Did not include a baseline assessment of diagnostic performance, so cannot directly quantify the  impact of NAT on learning. Third, because this was a study nested  within a real-world curriculum, they were limited on the manipulations  that could be performed, and the participants were free to use the  learning resources as much or as little as they wished. Fourth, there  were missing data that might have helped identify additional explanatory variables, such as skin tone, race, and ethnicity of participating  students. Fifth, the short duration of the study limits our ability the  distinguish a brief suppression of performance bias from learning. |
| Abeck 2024 | Pre-post intervention study (quasi-experimental design, no control group) | 158 medical students | Fourth-year in a six-year program | Mean: 24.2 years (range: 20–36) | F (101); M (55); Diverse (1) | Mandatory 90-minute seminar on skin type diversity, including interactive discussion and case-based learning | Standard curriculum, study was conducted at point when curriculum was 75% complete | Limited representation of skin diseases in patients with SoC in dermatology education | Significant increase in self-reported knowledge of dermatological conditions in SoC (pre: 2.9 ± 1.3, post: 4.5 ± 0.9, p < 0.001); ability to diagnose in SoC (pre: 2.3 ± 1.2, post: 3.9 ± 1.1, p < 0.001) | Students rated increased awareness of psychosocial burden of skin diseases in SoC (pre: 3.2 ± 1.3, post: 4.7 ± 0.9, p < 0.001) | Confidence in diagnosing skin diseases in SoC improved (p < 0.001) | Self-reported competence improved across all measured areas; 82.9% of students wanted more courses on this topic | NR | 0 | Students engaged in discussion about psychosocial burdens (e.g., PIH, vitiligo visibility); reinforced the need for SOC-specific dermatology education | This study is the first to evaluate a newly designed seminar on skin type diversity in an undergraduate medical curriculum in Germany. Lacks a control group and objective performance measures; long-term impact unknown. Response bias may be possible because of social desirability or the Hawthorne effect. |
| Peterknecht 2022 | Cross-sectional quality improvement study | 114 participants (39 medical students, 75 junior doctors, and 9 dermatology trainees) | Medical students and junior doctors (foundation doctors, internal medicine trainees, general practitioner trainees) | NR | NR | 60-minute interactive lecture covering SOC dermatology and a game-based activity for medical students | Single session per group, conducted at two NHS Trusts | Standard curriculum. 64% of students and 69% of junior doctors had received no training in Dermatology in SOC at undergraduate level. 84% of junior doctors had received no training at postgraduate level. | Confidence recognizing dermatological conditions in SOC improved (mean Likert score increase: students +2.50, junior doctors +1.82, p < 0.001); assessment scores improved (students +3.68, junior doctors +3.87, p < 0.001) | NR | Likert-scale survey (minimum score 1, maximum score 5) on participant confidence defining SOC, defining how inflammation may present in soc, recognizing common conditions in SOC, familiarity with conditions more prevalent in SOC, and diagnosing dermatoses in SOC | Confidence recognizing common conditions in SOC increased in medical students and junior doctors by 2.5 and 1.82. Increase in medical students’ confidence following game-based activity by 0.529. Mean assessment score for eight-question assessment also increased by 3.68 in medical students and 3.87 in junior doctors. | NR | NR | Participants highlighted gaps in SOC dermatology education and the need for curriculum updates | Study confirms inadequate SOC dermatology training; limited by sample size, single-session design, and lack of long-term follow-up |

**References**

1. Hijab E, Baranowski M, MacKelfresh J, Krueger L. A roadmap for the development of a diverse and inclusive medical student dermatologic curriculum. *J Drugs Dermatol.* 2022;21(8):908-910.
2. Peterknecht E, Strain J, Devereux C, et al. Improving recognition among undergraduate and postgraduate trainees of dermatological conditions in skin of colour: a cross-sectional quality-improvement project. *Clin Exp Dermatol.*2022;47(12):2261-2264.
3. Robinson JK, Mallett K, Wagner P, et al. Medical student detection of melanoma: clinical skills. *Arch Dermatol.*2010;146(10):1175-1177.
4. Abeck F, Schmidt S, Wurthmann S, et al. Effects of a skin-type diversity seminar on undergraduate medical students’ self-assessed competence in managing skin diseases in patients with skin of color. *BMC Med Educ.* 2024;24(1):848.
5. Arza A, Nguyen T, Smith L, et al. Beyond the surface: unveiling gaps in medical education through the eyes of diverse learners. *Arch Dermatol Res.* 2024;316(5):187-194.
6. Hardin J, Chen A, Shah N, et al. Mitigating diagnostic performance bias in a skin-tone balanced dermatology curriculum. *Clin Teach.* 2023;20(6):e13613.
7. Thompson B, Jenkins T, Sánchez M. Melanoma: does it present differently in darker skin tones? *MedEdPORTAL.*2023;19:11311.
8. Ibraheim MK, Mufti A, Plaster MJ, et al. Evaluating skin-of-color education in dermatology residency programs: data from a national survey. *Clin Dermatol.* 2022;40(2):228-233.
