# Supplementary material for: Training Medical Students/Medical Practitioners to Diagnose Dermatologic Conditions in Skin of Color: A Systematic Review of Educational Interventions
Source: J Cutan Med Surg. 2026 Jan 22;30(4):405–8. doi: 10.1177/12034754251408403 (PMC13478447; doi:10.1177/12034754251408403)
Supplement: Supplementary file 4 [file sj-docx-4-cms-10.1177_12034754251408403.docx]

**Supplementary Table 3.** Different intervention of teaching style and its key results.

| **Intervention**  **(%, n/N)** | **Study type**  **(%, n/N)** | **Level of Education** | **Duration of Intervention, weeks (n/N)** | **Knowledge Gain Measurement** | **Cultural Competence** | **Confidence or Self-Efficacy** | **Key Results** | **Qualitative Finding** |
| --- | --- | --- | --- | --- | --- | --- | --- | --- |
| **Didactic Teaching (Lecture-Based learning) (33.1%, 221/667)** | | | | | | | | |
| Lecture-based Curriculum (Hijab)  (48.4%, 107/221) | Descriptive | First-year medical students | 1 week | Pre-post-test | Likert-scale surveys | Self-reported Survey | Statistically significant increase in view of dermatology as a culturally competent specialty (P<0.001), increase in interest in dermatology (P=0.048) | Students preferred learning about disease variations across populations |
| 60 minutes interactive lecture covering SOC (Peterknecht)  (51.6%, 114/221) | Cross-sectional quality improvement study | Medical Students + Junior Doctors | 1 session of 60 mins (<1 week) | Eight question assessment | NR | Likert-scale survey | Confidence recognizing common conditions in SOC increased by 2.5 and 1.82 in medical students and junior doctors respectively (p < 0.001). Increase in medical students’ confidence following game-based activity by 0.529. Mean assessment score for eight-question assessment increased by 3.68 and 3.87 in medical students and junior doctors respectively (p < 0.001). | Participants highlighted gaps in SOC dermatology education and the need for curriculum updates |
| **Seminar-Style Expert-Led Teaching (32.4%, 216/667)** | | | | | | | | |
| Virtual Dermatologist-led Seminar (Arza)  (26.9%, 58/216) | Quasi-experimental | 1st to 4th year Medical Students | 1 session (<1 week) | Pre-post test | Likert-scale | Post-seminar survey | 90% reported lower confidence diagnosing skin conditions on darker vs. lighter skin. After the seminar, 87.2% still lacked confidence diagnosing SOC conditions, with 78.7% citing poor curriculum coverage. Confidence was significantly lower for MST 6–10 vs. MST 1–5 (p < 0.009). | Students criticized the Eurocentric bias in textbooks and lack of diverse faculty, calling for improved SOC representation in medical education |
| Mandatory 90-minute seminar and discussion and case-based learning (Abeck)  (73.1%, 158/216) | Quasi-experimental | 4th year MD students in 6 year program | 1 session of 90 mins (<1 week) | Pre-post test | Likert-scale | Likert-scale | Self-assessed knowledge of SOC skin disease presentations increased from 2.9 ± 1.3 to 4.5 ± 0.9 (t(157) = −4.158, p < 0.001). Perceived relevance of SOC dermatology increased from 5.2 ± 1.2 to 5.6 ± 0.7 (t(157) = −14.610, p < 0.001). Students rated increased awareness of psychosocial burden of skin diseases in SoC from 3.2 ± 1.3, to 4.7 ± 0.9 (p < 0.001) and their ability to diagnose in SOC increased from 2.3 ± 1.2 to 3.9 ± 1.1, (p < 0.001). Self-reported competence improved across all measured areas (p < 0.001); 82.9% of students wanted more courses on this topic | Students engaged in discussion about psychosocial burdens (e.g., PIH, vitiligo visibility); reinforced the need for SOC-specific dermatology education |
| **Interactive/ Workshop-Based Learning (25.8%, 172/667)** | | | | | | | | |
| Interactive Workshop (Powerpoint/ Video/ Reflection/ Case studies) (Thompson)  (46.5%, 80/172) | Quasi-experimental | Medical students, residents and nursing students | 1 session of 60 mins (<1 week) | Pre-post test | NR | Likert Scale, Case-based discussion + video-based reflection | Statistically significant increase in learners’ self-reported confidence across all learning objectives | Participants valued interactive and multi-modal learning. Requested more SOC images and better slide readability. |
| Online non-analytic training with Workshop (Hardin)  (53.5%, 92/172) | Prospective randomized | 1st Year Medical Students | 2 weeks | Pre-post test | Surveys | Likert Scale | After NAT (week 1), students performed worse on SoC cases (*p* = 0.0002 for LS-trained, *p* = 0.002 for SoC-trained). After CT (week 2), diagnostic bias was eliminated (*p* = 0.0001 for LS-trained, *p* = 0.007 for SoC-trained). Post-CT, SoC and LS performance were equal, though diagnostic accuracy for melanoma on SoC remained lower than on LS, indicating residual condition-specific gaps. | Before starting the dermatology course, the majority of students did not realise that there were challenges related to diagnosing some skin conditions in SoC. |
| **Simulation-Based Learning (8.7%, 58/667)** | | | | | | | | |
| Opportunistic Screening Exercise using Moulages (Robinson)  (100%, 58/58) | Descriptive (clinical observation) | 2nd Year Medical Students | 1 session of 60 mins (<1 week) | Observational detection exercise | NR | NR | Only 16% of students detected the melanoma moulage. Equal detection on Black vs. white patients, but students were more likely to suggest melanoma in white patients (p = 0.03). Students interested in dermatology were more likely to notice lesions (p = 0.04). | Participants valued interactive and multi-modal learning. Requested more SOC images and better slide readability. |
